# Supplementary figures and images for: Validation of a Novel Fluorescent Lateral Flow Assay for Rapid Qualitative and Quantitative Assessment of Total Anti-SARS-CoV-2 S-RBD Binding Antibody Units (BAU) from Plasma or Fingerstick Whole-Blood of COVID-19 Vaccinees
Source: Vaccines (Basel). 2022 Aug 15;10(8):1318. doi: 10.3390/vaccines10081318 (PMC9415525; doi:10.3390/vaccines10081318)

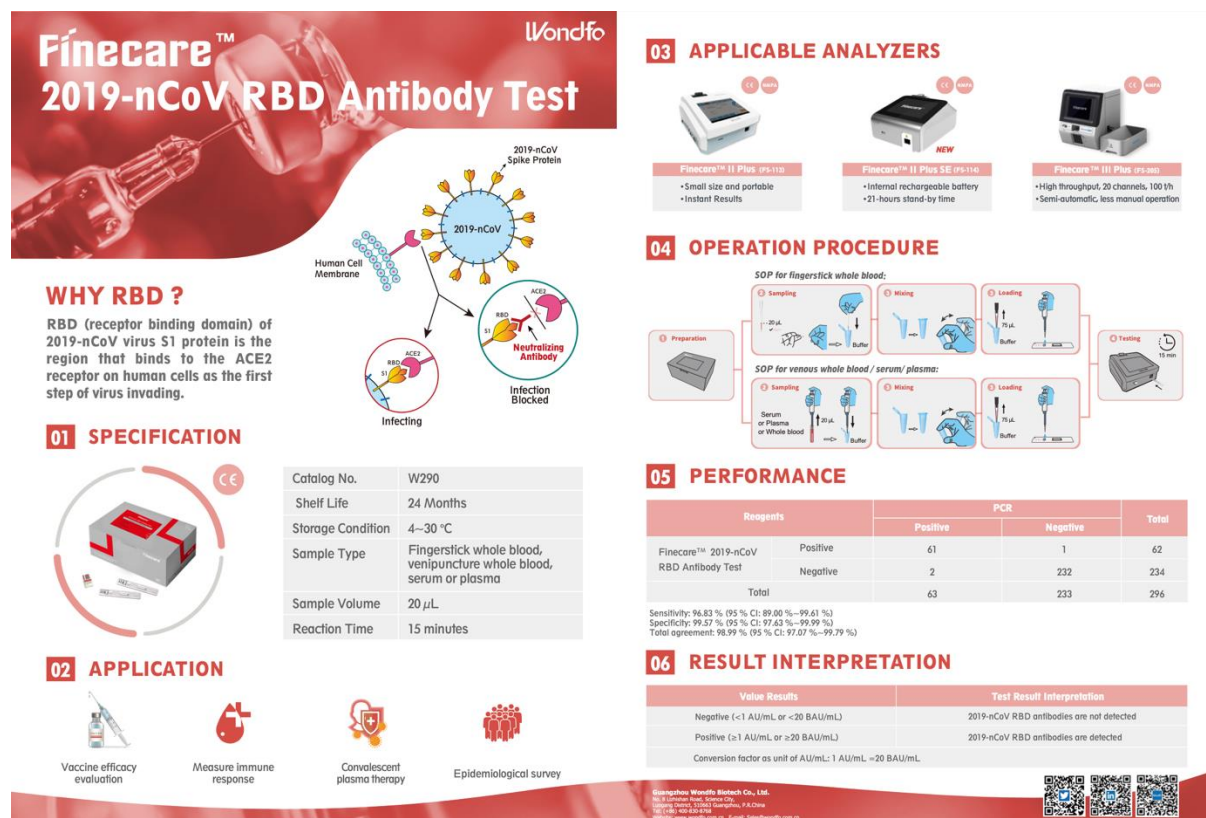

Supplement: Supplementary file 1 [file vaccines-10-01318-s001.zip › vaccines-1844408-supplementary.pdf]
